# Temperature-Dependent Microstructure Evolution and Superplastic Deformation Behavior of Cold-Deformed Cr4Mo4Ni4V Martensitic Steel: From Continuous to Discontinuous Dynamic Recrystallization

**DOI:** 10.3390/ma19112242

**Published:** 2026-05-26

**Authors:** Jiwei Wang, Wanli Yang, Jiabin Liu, Tao Li, Wei Tang, Bin Shao, Yingying Zong

**Affiliations:** 1 AECC Shenyang Liming Aero-Engine Co., Ltd., Shenyang 110043, China; 2 National Key Laboratory for Precision Hot Processing of Metals, Harbin Institute of Technology, Harbin 150001, China

**Keywords:** Cr4Mo4Ni4V martensitic steel, superplasticity, dynamic recrystallization, continuous dynamic recrystallization, discontinuous dynamic recrystallization

## Abstract

To elucidate the evolution of dynamic recrystallization (DRX) mechanisms in cold-worked Cr4Mo4Ni4V martensitic steel, tensile tests were conducted on a 50% cold-deformed material at 600–850 °C at a fixed strain rate of 0.001 s^−1^, combined with systematic microstructural characterization. Under this specific strain rate, the results reveal a temperature-dependent transition from continuous dynamic recrystallization (CDRX) to discontinuous dynamic recrystallization (DDRX). At 600 °C, CDRX dominates, producing recrystallized grains with orientations close to the parent matrix and relatively strong texture. At 750 °C, CDRX and DDRX coexist, while DDRX is significantly enhanced, characterized by grain boundary nucleation and random orientations, leading to a marked reduction in texture intensity; simultaneously, the fraction of recrystallized grains and high-angle grain boundaries reaches a maximum. At 850 °C, DDRX becomes dominant. This transition in DRX mechanism governs the high-temperature plasticity, with optimal superplasticity achieved at 800 °C, corresponding to an elongation of 748%. Cavities are primarily initiated at carbide/matrix interfaces, and their growth and coalescence dominate the fracture process. These findings clarify the temperature-dependent DRX evolution and its role in regulating superplasticity, providing guidance for microstructure design and superplastic forming of martensitic steels.

## 1. Introduction

Superplastic forming has emerged as a critical processing route in advanced manufacturing owing to its exceptional elongation, high material utilization, low flow stress, and excellent dimensional accuracy for large and complex components, and has therefore attracted extensive attention in aerospace and automotive industries [1,2,3,4].

Dynamic recrystallization (DRX) plays a pivotal role in governing high-temperature plastic deformation, exerting a decisive influence on grain refinement, microstructural evolution, and flow stability during superplastic deformation [5,6]. Depending on the nucleation mechanism, DRX is generally classified into continuous dynamic recrystallization (CDRX) and discontinuous dynamic recrystallization (DDRX) [7,8], both of which promote grain refinement, increase the fraction of high-angle grain boundaries, and reduce dislocation density [9]. Considerable efforts have been devoted to understanding DRX mechanisms during superplastic deformation. For example, Zou et al. [10] reported that in Al–Cu–Li alloys, subgrain rotation and coalescence lead to equiaxed recrystallized grains, where CDRX cooperates with grain boundary sliding, while the Zener–Hollomon parameter strongly affects the competition between dynamic recovery and recrystallization. Zhang et al. [11] demonstrated that in medium-Mn steels, temperature-dependent coupling between DRX and deformation-induced phase transformation enables grain boundary sliding, resulting in elongations exceeding 1500% at 800 °C. Li et al. [12] revealed that in Ti60 alloy, DRX acts as the dominant softening mechanism, where CDRX and DDRX coexist at early stages but progressively transition to DDRX-dominated behavior with increasing strain. Among various factors, deformation temperature is one of the most critical parameters governing DRX behavior [13]. However, a systematic understanding of the temperature-dependent evolution of DRX mechanisms, particularly the competition and transition between CDRX and DDRX and their influence on superplastic deformation behavior, remains insufficiently established.

Cold-worked martensitic steels exhibit unique advantages for achieving superplasticity via DRX [14]. The intrinsic characteristics of martensitic microstructures, including refined lath structures, high dislocation density, and supersaturated carbon [15], facilitate the formation of ultrafine grains during subsequent deformation, thereby enabling superplastic deformation [16,17]. It has been reported that lath martensite can evolve into ultrafine-grained structures with superplasticity under thermomechanical processing [18,19], and even appropriately designed martensitic steels may exhibit superplastic behavior without complex processing routes [20]. Moreover, moderate cold deformation has been shown to produce elongations approaching 700%, where the synergistic interaction between CDRX and DDRX promotes grain boundary sliding [21]. Nevertheless, the influence of deformation temperature on DRX mechanism evolution in cold-worked martensitic steels during superplastic deformation remains unclear.

Cr4Mo4Ni4V, a high-alloy low-carbon steel and a typical high-temperature bearing steel, belongs to the martensitic steel family and is widely used in critical components of aero-engines and high-temperature forming dies due to its excellent mechanical properties [22,23]. Such components often possess complex geometries, imposing stringent requirements on superplastic forming capability and microstructural control [24,25].

In this context, the present study systematically investigates the superplastic deformation behavior of cold-worked Cr4Mo4Ni4V martensitic steel via high-temperature tensile tests in the temperature range of 600–850 °C. The microstructural evolution is comprehensively characterized, with particular emphasis on the temperature-dependent transition of DRX mechanisms and their role in governing superplasticity. This work aims to clarify the evolution of CDRX and DDRX with temperature and their competitive relationship, providing fundamental insights for optimizing superplastic forming processes in martensitic steels [26].

## 2. Materials and Experiment

**Figure 1:**
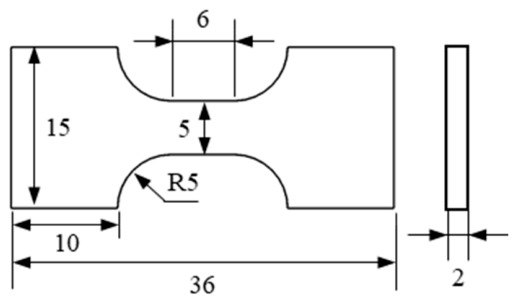
Dimensions of tensile specimens (in mm).

The chemical composition of the Cr4Mo4Ni4V steel used in this study is listed in Table 1. Cylindrical specimens with a diameter of 50 mm and a height of 30 mm, in the spheroidization-annealed condition, were heated to 1050 °C and held for 0.5 h, followed by 50% compression along the height direction. The deformed specimens were then air-cooled to room temperature. Subsequently, a secondary compression with a reduction of 50% was performed at room temperature. Dog-bone-shaped tensile specimens with dimensions of 6 mm × 5 mm × 2 mm were machined from the central region, the Dimensions of tensile specimens as shown in Figure 1. High-temperature tensile tests were conducted in the temperature range of 600–850 °C at a strain rate of 0.001 s^−1^. After reaching the target temperature, the specimens were held in a high-temperature furnace for 0.5 h to ensure thermal homogenization. Immediately after deformation, water quenching was applied to preserve the deformed microstructure.

An SU 5000 scanning electron microscope (SEM) (Hitachi High-Tech Corporation, Tokyo, Japan) mounted with an EDAX-TSL electron backscatter diffraction (EBSD) system (EDAX, Mahwah, NJ, USA) and an EDAX energy dispersive X-ray spectrometer (EDS) were used for SEM observation, Specimens for scanning electron microscopy (SEM) were prepared using standard metallographic procedures. After mechanical grinding and polishing, the samples were etched in a solution of 10 vol.% nitric acid in ethanol for 30 s. For electron backscatter diffraction (EBSD) analysis, mechanically polished specimens were further electropolished at approximately −20 °C in a solution of 10 vol.% perchloric acid for about 40 s. The precipitated phase composition and morphology were analyzed by FEI Talos 200X Transmission Electron Microscope (TEM) (FEI, Hillsboro, OR, USA) equipped with a high angle annular dark field (HAADF) detector. Specimens were mechanically thinned to a thickness of 50–70 μm and punched into 3 mm disks. Final thinning was carried out using a twin-jet electropolishing system in a solution of 10 vol.% perchloric acid at approximately −20 °C and 20 V until electron transparency was achieved.

## 3. Results and Discussion

### 3.1. High-Temperature Tensile Properties

**Figure 2:**
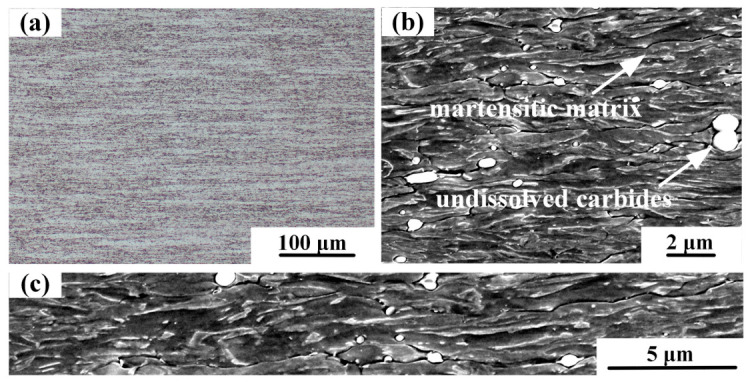
Multiscale microstructural features of the 50% cold-compressed martensitic specimen. (**a**) Boundary network; (**b**) martensite–carbide composite structure; (**c**) elongated martensitic blocks.

Figure 2 shows the microstructural features of the 50% cold-compressed martensitic specimen at different length scales. After cold deformation, a pronounced lamellar structure develops along the compression direction in the matrix. Although prior austenite grain boundaries are not clearly resolved in local regions, the prior austenite grain boundaries, packet boundaries, and martensite block boundaries are all observed as continuous dark line features in Figure 2a. Due to the preferential removal of the matrix during specimen preparation or etching, the carbides protrude slightly from the matrix surface, thus appearing white in SEM [28,29]. The SEM image (Figure 2b) reveals a composite microstructure consisting of a martensitic matrix and undissolved carbides. The carbides are homogeneously dispersed within the matrix, exhibiting an equiaxed morphology, and show no obvious deformation or fragmentation during cold compression. At higher magnification (Figure 2c), a typical elongated martensitic block structure is observed, with a pronounced elongation and alignment along the deformation direction.

**Figure 3:**
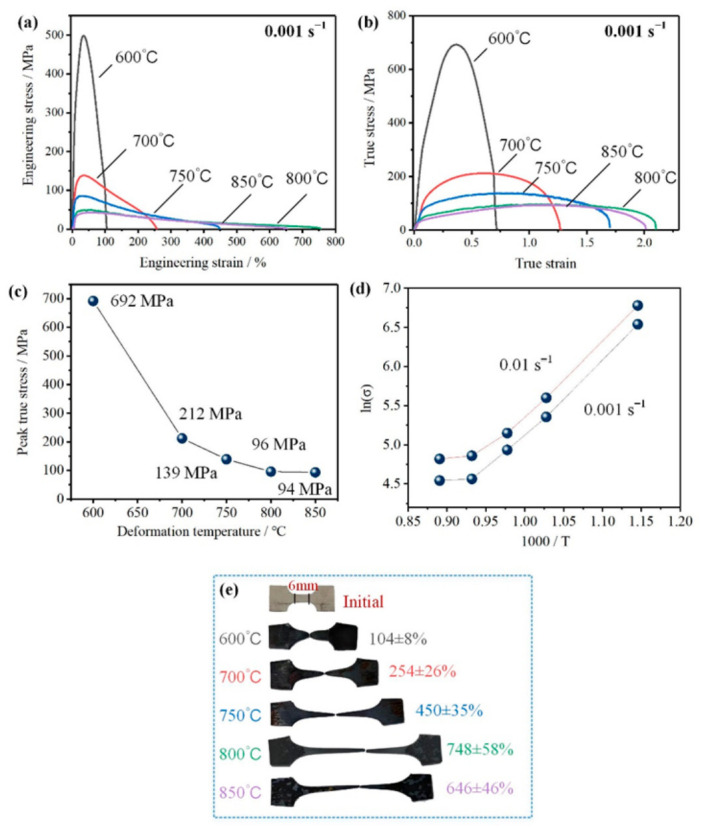
Tensile stress–strain curves and macroscopic morphologies of the cold-deformed martensitic steel at different deformation temperatures. (**a**) Engineering stress–strain curves; (**b**) true stress–strain curves; (**c**) variation in peak true stress with deformation temperature; (**d**) stress temperature–strain rate curves; (**e**) macroscopic appearance of tensile specimens after deformation.

Figure 3 shows the stress–strain responses obtained from high-temperature tensile tests conducted in the temperature range of 600–850 °C at a strain rate of 0.001 s^−1^. Engineering stress–strain curves are presented in Figure 3a, while true stress–true strain curves are shown in Figure 3b. At 600 °C, the specimen exhibits a limited elongation of 104%, with a peak true stress of 692 MPa, indicating high strength but poor ductility. With increasing deformation temperature, dynamic recrystallization-induced softening becomes progressively more pronounced, leading to a continuous decrease in peak true stress and a significant improvement in elongation. At 700 °C, the peak true stress decreases sharply to 212 MPa, while the elongation increases to 254%, indicating enhanced plasticity. When the temperature is further increased to 750 °C, the elongation reaches 450%, demonstrating improved high-temperature ductility. At 800 °C, the peak stress drops below 100 MPa, and the elongation reaches a maximum of 748%, reflecting a pronounced superplastic response. As reported by Yang et al. [21], under the same degree of compression, the elongation of cold-deformed martensite samples is significantly higher than that of cold-deformed ferrite samples. At 800 °C and a strain rate of 0.001 s^−1^, the elongation of the cold-deformed ferrite samples reaches only 500%.

However, when the deformation temperature is increased to 850 °C, grain coarsening occurs, resulting in a reduction in elongation to 646%. Although elevated temperatures promote plastic flow, excessive grain growth limits further improvement in ductility. The variation in peak stress with deformation temperature and stress temperature–strain rate is shown in Figure 3c,d, while the corresponding macroscopic appearances of the specimens are shown in Figure 3e.

The superplastic deformation mechanism can be assessed through the strain rate sensitivity index (m), determined via SRJ tests:

(1) $$m=\frac{\ln(\sigma_{1}/\sigma_{2})}{\ln({\dot{\varepsilon }}_{1}/{\dot{\varepsilon }}_{2})}$$

in which σ_1_ and σ_2_ are the stress before or after the jump at the point of rate change at ${\dot{\varepsilon }}_{1}$ or ${\dot{\varepsilon }}_{2}$, respectively. The values of m at different temperatures as shown in Table 2.

Additionally, for a more comprehensive illustration of the superplastic deformation mechanisms of Cr4Mo4Ni4V steel, the deformation activation energy (Q) is calculated using Equation (2):

(2) $$Q=\frac{1}{m}\cdot R\cdot \frac{\partial \ln\sigma }{\partial \left(\frac{1}{T}\right)}{|}_{\dot{\varepsilon }}$$

in which T and R represent the absolute temperature and the universal gas constant (8.314 J mol^−1^ K^−1^), respectively. Calculated deformation activation energies as shown in Table 3.

### 3.2. Microstructure Evolution

**Figure 4:**
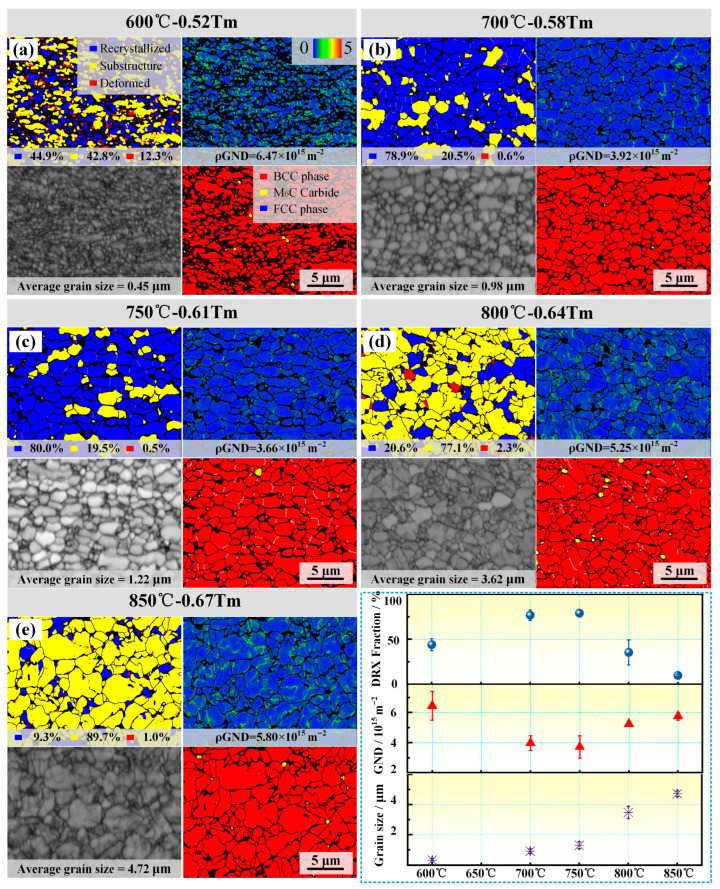
Recrystallization and KAM maps of tensile specimens of cold-deformed martensitic steel at different deformation temperatures. (**a**–**e**) Microstructures in the uniform elongation region at 600–850 °C.

Figure 4 shows the microstructures in the uniform elongation region of tensile specimens deformed in the temperature range of 600–850 °C. With increasing deformation temperature, the fraction of recrystallized grains first increases and then decreases. As shown in Figure 4a, at 600 °C, the recrystallized grain fraction is 44.9%. It increases to 78.9% at 700 °C (Figure 4b) and reaches a maximum of 80% at 750 °C (Figure 4c). At higher temperatures (Figure 4d,e), the recrystallized grain fraction decreases, while the fraction of recovered grains increases. This transition can be attributed to the significant increase in elongation, during which dislocation activity plays a more dominant role in accommodating plastic deformation. The grain size increases continuously with deformation temperature. At 600 °C, the average grain size is only 0.45 μm. Using martensite as the starting microstructure can effectively reduce the final grain size compared to ferrite. This advantage arises from the higher dislocation density and the further subdivided hierarchical structures (e.g., lath, block, and packet boundaries) inherent in martensite. During high-temperature deformation, these abundant dislocations and internal interfaces serve as preferential nucleation sites for dynamic recrystallization (DRX), leading to a higher nucleation rate in martensite than in ferrite. In contrast, the ferrite starting structure, with its lower dislocation density and coarser initial grain size, requires larger deformation strains to achieve comparable grain refinement. Consequently, the martensitic initial state enables a more efficient reduction in grain size under the same deformation conditions. The most pronounced grain growth occurs when the temperature increases from 750 °C to 800 °C, where the average grain size increases from 1.22 μm to 3.62 μm. This grain coarsening is governed by the combined effects of increasing deformation temperature and prolonged deformation time.

**Figure 5:**
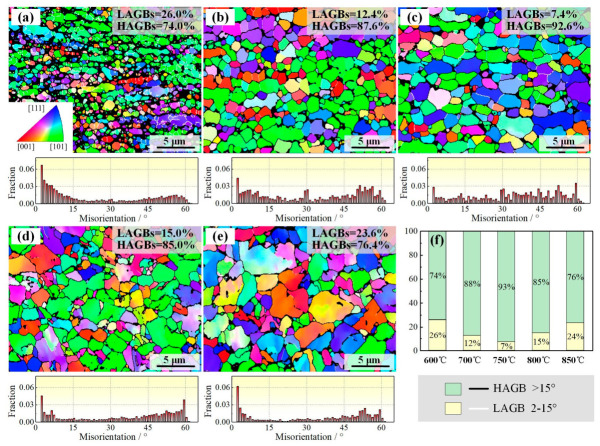
Microstructures of cold-deformed martensitic steel specimens deformed at different temperatures. (**a**–**e**) Inverse pole figure (IPF) maps at 600–850 °C; (**f**) evolution of high-angle and low-angle grain boundary fractions with deformation temperature.

Figure 5 shows inverse pole figure (IPF) maps and misorientation distributions of tensile specimens in the uniform elongation region deformed in the temperature range of 600–850 °C. The fraction of high-angle grain boundaries (HAGBs) exhibits a trend consistent with that of the recrystallized grain fraction, reaching a maximum of 93% at 750 °C. In contrast, the HAGB fraction remains close to ~75% at both 600 °C and 850 °C.

**Figure 6:**
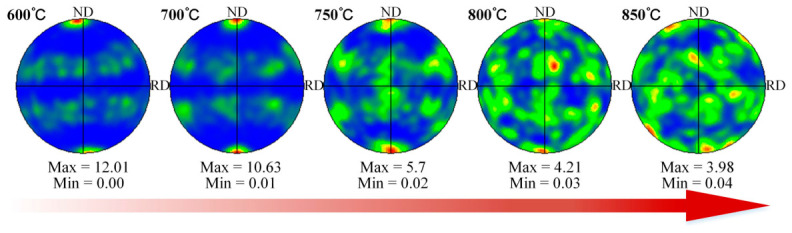
Texture evolution of cold-deformed martensite specimens deformed at different temperatures.

Figure 6 shows the evolution of {111} pole figures with deformation temperature. At 600 °C, a pronounced <110> ∥ RD deformation texture is observed, with a maximum texture intensity of 12.01, which is similar to that typically reported in cold-drawn BCC wires. With increasing deformation temperature, discontinuous dynamic recrystallization becomes progressively more dominant, leading to a more randomized orientation distribution of newly formed recrystallized grains. The increasing misorientation between recrystallized and parent grains promotes a more homogeneous microstructure and suppresses the development of strong texture. In addition, the combined effects of higher elongation, longer deformation time, grain growth, and grain rotation further weaken the texture intensity. At 850 °C, the texture intensity decreases significantly to 3.98.

**Figure 7:**
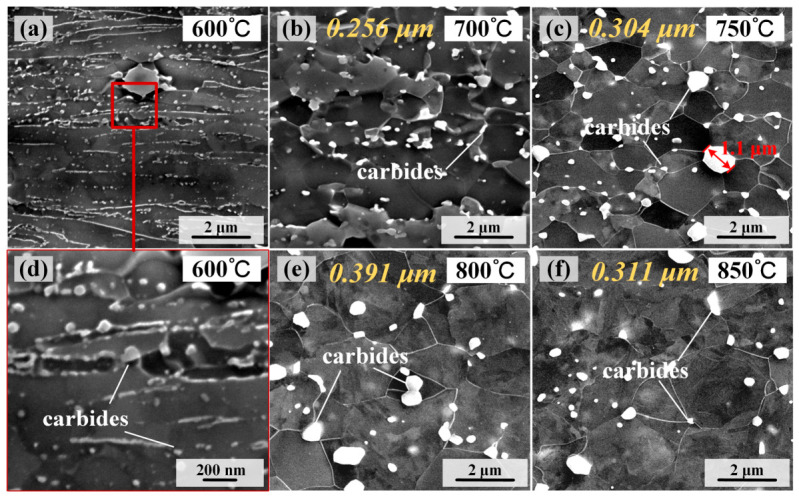
Carbide morphology and distribution in cold-deformed martensitic steel specimens deformed at different temperatures: (**a**) 600 °C; (**b**) 700 °C; (**c**) 750 °C; (**d**) magnified view of the red rectangular region in (**a**); (**e**) 800 °C; (**f**) 850 °C. Above the image is the average size of the carbides.

Figure 7 shows secondary electron SEM images of tensile specimens in the uniform elongation region deformed in the temperature range of 600–850 °C. At 600 °C (Figure 7a), numerous nanoscale carbides are precipitated in the matrix, while no clear evidence of recrystallization nucleation is observed. At 700 °C (Figure 7b), a mixed microstructure consisting of banded grains and equiaxed grains is formed. A large number of recrystallized grains nucleate and bulge along grain boundaries, while carbides coarsen and are mainly distributed along grain boundaries. On the basis of the grain refinement achieved through cold-deformed martensite, the carbides located along grain boundaries effectively pin the boundaries, which helps maintain a stable grain size during high-temperature deformation. This grain boundary pinning effect is conducive to the occurrence of superplasticity. At 750 °C (Figure 7c), the degree of grain equiaxialization is significantly enhanced, indicating more extensive recrystallization. When the deformation temperature increases to 800 °C and 850 °C (Figure 7e,f), pronounced grain coarsening occurs, and the equiaxed grain size increases substantially.

**Figure 8:**
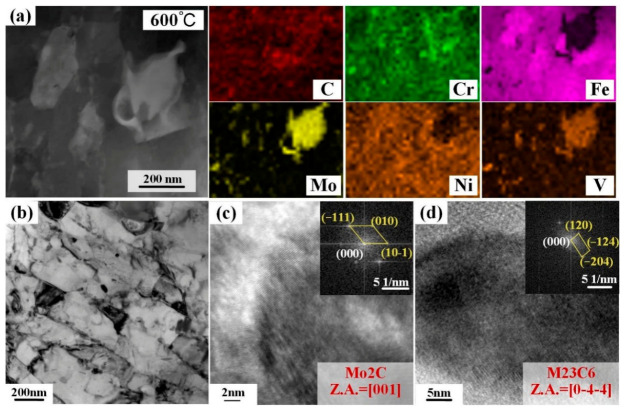
Types of precipitated carbides in cold-deformed martensitic steel deformed at 600 °C. (**a**) Elemental mapping; (**b**) bright-field image; (**c**,**d**) high-resolution characterization of nanoscale carbides.

Figure 8 shows TEM characterization of nanoscale carbides dynamically precipitated during deformation at 600 °C. Elemental mapping in Figure 8a reveals enrichment of C, Cr, Mo, and V, together with Fe depletion, which is characteristic of alloy carbide precipitation. The bright-field image (Figure 8b) indicates the formation of submicron grains with sizes of 200–500 nm, generated through the subdivision of martensitic laths. As shown in Figure 8c,d, high-resolution imaging combined with fast Fourier transform (FFT) analysis confirms that the nanoscale precipitates consist of multiple carbide types, including Mo_2_C and M_23_C_6_.

### 3.3. Fracture Surface Characterization

**Figure 9:**
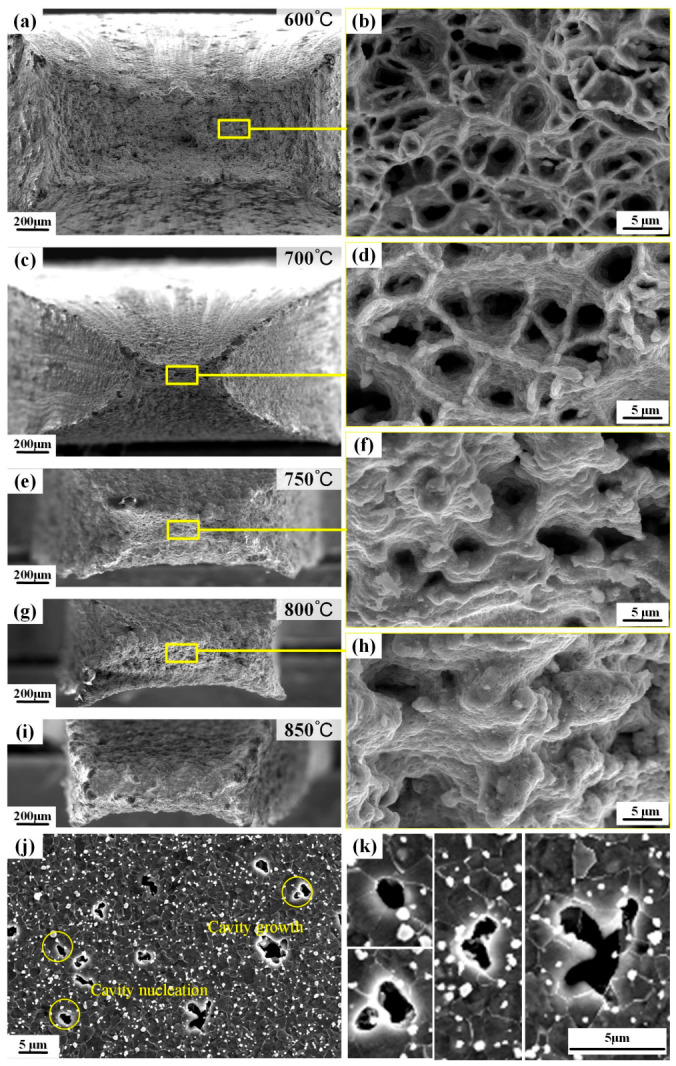
Fracture morphology and near-fracture microstructure of cold-deformed martensitic steel at different temperatures: (**a**,**b**) 600 °C; (**c**,**d**) 700 °C; (**e**,**f**) 750 °C; (**g**,**h**) 800 °C; (**i**) 850 °C; (**j**,**k**) cross-section near the fracture of the 800 °C specimen.

Figure 9 shows the fracture morphologies and voids near the fracture surfaces of cold-deformed martensitic steel specimens deformed at different temperatures. At relatively low deformation temperatures (Figure 9a–d), the fracture surfaces exhibit a distinct necking region, and the central fracture area is characterized by fine, equiaxed dimples, indicating ductile fracture. When the deformation temperature increases to 700 °C, the dimple size increases, corresponding to an elongation of 254% and a pronounced increase in reduction in area. At temperatures ≥ 750 °C (Figure 9e–i), the specimens exhibit typical superplastic deformation behavior, characterized by homogeneous thinning without obvious necking. Corresponding to elongations exceeding 400%, a large number of voids are observed in the fracture center. Secondary electron images of the specimen deformed at 800 °C (Figure 9j,k) reveal that voids preferentially nucleate at the carbide/matrix interfaces. The nucleation, growth, and coalescence of these voids ultimately lead to fracture.

### 3.4. Evolution of DRX Mechanisms

**Figure 10:**
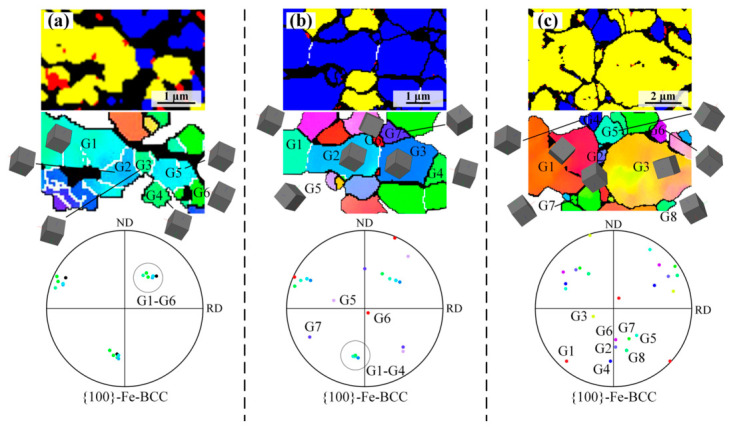
Recrystallized grain orientation analysis of specimens deformed at various temperatures: (**a**) 600°C; (**b**) 750°C; (**c**) 850°C.

Figure 10 shows the orientation characteristics of recrystallized grains at different deformation temperatures, revealing a systematic transition of the dynamic recrystallization (DRX) mechanism from continuous dynamic recrystallization (CDRX) to discontinuous dynamic recrystallization (DDRX) with increasing temperature. This transition is closely associated with the high-temperature plasticity of the material and is critical for understanding the superplastic deformation behavior of cold-deformed Cr4Mo4Ni4V martensitic steel.

At 600 °C, recrystallized grains exhibit negligible misorientation relative to the parent matrix and undergo only slight lattice rotation, indicating a typical CDRX-dominated mechanism. As the deformation temperature increases to 750 °C, CDRX and DDRX coexist. As shown in Figure 10b, grains G1–G4 exhibit characteristics of CDRX, with orientations similar to the parent matrix, whereas grains G5–G7 show significant misorientation, nucleating and growing at grain boundaries with small grain sizes, corresponding to DDRX. At this temperature, the recrystallized grain fraction reaches a maximum of 80%, accompanied by a peak in the high-angle grain boundary fraction (93%). The activation of DDRX indicates that prior grain boundaries act as effective nucleation sites, where new grains form via grain boundary bulging. The randomly distributed orientations of these newly formed grains significantly weaken the deformation texture. The synergistic operation of CDRX and DDRX promotes grain boundary sliding and maintains a stable microstructure through continuous replenishment of fine equiaxed grains, resulting in a substantial increase in elongation to 450%. Notably, CDRX remains active at this stage, indicating a coupled and complementary relationship between the two mechanisms. When the deformation temperature is further increased to 800–850 °C, DDRX becomes the dominant mechanism (Figure 10c). Recrystallized grains are randomly oriented and predominantly located at grain boundaries, corresponding to a very low texture intensity. With increasing temperature, the contribution of CDRX decreases while DDRX becomes increasingly dominant.

The influence of DRX mechanism transition on superplastic deformation can be summarized in three aspects: (i) at 600 °C, CDRX dominates, leading to limited grain refinement and relatively low ductility; (ii) at 750 °C, the coexistence of CDRX and DDRX produces peak recrystallized fraction and high-angle grain boundary fraction, resulting in significantly enhanced elongation; and (iii) at 800–850 °C, DDRX dominates, which effectively weakens texture, but grain coarsening at high temperature limits further improvement in ductility. Notably, although the recrystallized grain fraction does not reach its maximum at 800 °C, the highest elongation (748%) is achieved, indicating that superplasticity is not solely governed by recrystallized fraction, but also strongly dependent on grain boundary sliding compatibility and void suppression capability.

## 4. Conclusions

This study investigates the superplastic behavior and microstructure evolution of cold-deformed martensitic Cr4Mo4Ni4V steel at different deformation temperatures. The main conclusions are as follows:

(1) At the strain rate of 0.001 s^−1^, the elongation of the cold-deformed martensitic steel first increases and then decreases with increasing deformation temperature from 600 °C to 850 °C, reaching a maximum of 748% at 800 °C. Fracture is primarily governed by void nucleation at carbide/matrix interfaces, followed by void growth and coalescence.
(2) With increasing deformation temperature from 600 °C to 850 °C, the fraction of recrystallized grains first increases and then decreases, reaching a maximum of 80% at 750 °C, accompanied by a peak high-angle grain boundary fraction of 93%. The average grain size increases from 0.45 μm to 3.62 μm, while the texture intensity decreases markedly from 12.01 to 3.98, indicating an enhanced capability of recrystallization to eliminate the deformation-induced texture with increasing temperature.
(3) At the strain rate of 0.001 s^−1^, the dynamic recrystallization mechanism evolves from CDRX to DDRX with increasing temperature. At 600 °C, CDRX dominates with a relatively low recrystallized fraction of 44.9%, providing limited softening. At 750 °C, CDRX and DDRX operate synergistically, leading to a peak recrystallized fraction of 80% and a corresponding high-angle grain boundary fraction of 93%. At 850 °C, DDRX becomes dominant, with recrystallized grains nucleating at grain boundaries and exhibiting random orientations.
(4) Under the strain rate of 0.001 s^−1^, the optimum superplastic forming window for cold-deformed martensitic Cr4Mo4Ni4V steel is 750–800 °C. In this range, the synergistic operation of CDRX and DDRX can be effectively exploited, ensuring continuous microstructural refinement and thus promoting superplastic deformation.

## Figures and Tables

**Table 1:** Chemical composition of Cr_4_Mo_4_Ni_4_V steel (wt%) [27].

C	Cr	Mo	V	Ni	Mn	Si	Fe
0.13	4.12	4.25	1.23	3.40	0.20	0.12	Bal.

**Table 2:** Calculated m values.

Temperature	600	700	750	800	850
m	0.10	0.23	0.3	0.33	0.32

**Table 3:** Calculated deformation activation energies.

Temperature/°C	600	700	750	800	850
Q/kJ mol^−1^	804.24	292.91	222.9	195.2	128.4

## Data Availability

The original contributions presented in the study are included in the article, further inquiries can be directed to the corresponding author.

## References

[1] Masuda H., Sato E. (2020). Diffusional and dislocation accommodation mechanisms in superplastic materials. Acta Mater..

[2] Song Z., Niu R., Cui X., Bobruk E.V., Murashkin M.Y., Enikeev N.A., Gu J., Song M., Bhatia V., Ringer S.P. (2023). Mechanism of room-temperature superplasticity in ultrafine-grained Al–Zn alloys. Acta Mater..

[3] Venkatesh B., Panigrahi S.K. (2024). A superplastic micro-extrusion technology to develop engineered magnesium micro-components. J. Mater. Process. Technol..

[4] Nazeer F., Long J., Yang Z., Li C. (2022). Superplastic deformation behavior of Mg alloys: A review. J. Magnes. Alloy..

[5] Jia Y., Wu S., Mu Y., Xu L., Ren C., Sun K., Yi J., Jia Y., Yan W., Wang G. (2023). Efficient Coarse-Grained Superplasticity of a Gigapascal Lightweight Refractory Medium Entropy Alloy. Adv. Sci..

[6] Wang H., Koenigsmann K., Zhang S., Li Y., Liu H., Liu H., Ren L., Qiu D., Yang K. (2023). Extraordinary superplasticity at low homologous temperature and high strain rate enabled by a multiphase nanocrystalline network. Int. J. Plast..

[7] Yin L., Sun Z., Fan J., Yin Z., Wang Y., Dang Z. (2024). Dynamic recrystallization in a near β titanium alloy under different deformation modes–Transition and correlation. Acta Mater..

[8] Yang X., Chen S., Wang B., Li X., Wang B., Tian Y. (2022). Superplastic deformation behavior of cold-rolled Inconel 718 alloy at high strain rates. J. Mater. Process. Technol..

[9] Eleti R.R., Bhattacharjee T., Shibata A., Tsuji N. (2019). Unique deformation behavior and microstructure evolution in high temperature processing of HfNbTaTiZr refractory high entropy alloy. Acta Mater..

[10] Zou G., Zhang R., Wang W., Li J., Ye L. (2025). Understanding effects of deformation parameters on dynamic recrystallization-dependent superplasticity in an Al-Cu-Li alloy. Mater. Des..

[11] Zhang H.T., Xiao N., Sun S.H., Gao X.K., Yan H.L., Cai M.H. (2025). Unraveling temperature-dependent superplastic deformation and microstructure evolution in warm-rolled Fe-10Mn-4Al-1.5Si-0.3C medium Mn steel. Mater. Sci. Eng. A.

[12] Li Y., Jiang S., Peng P., Zhang J., Yang S., Lu Z., Jia Y. (2025). Superplastic deformation and macrozone behavior of the Ti60 high-temperature titanium alloy. J. Alloy. Compd..

[13] Alabort E., Kontis P., Barba D., Dragnevski K., Reed R.C. (2016). On the mechanisms of superplasticity in Ti–6Al–4V. Acta Mater..

[14] Pan H., Li X., Zhang H., Liu L., Wu Z. (2023). Achieving low-temperature superplasticity in a cold-rolled medium Mn steel with an equilibrium ultrafine equiaxed dual-phase microstructure. Mater. Sci. Eng. A.

[15] Ueji R., Tsuji N., Minamino Y., Koizumi Y. (2002). Ultragrain refinement of plain low carbon steel by cold-rolling and annealing of martensite. Acta Mater..

[16] Poorganji B., Miyamoto G., Maki T., Furuhara T. (2008). Formation of ultrafine grained ferrite by warm deformation of lath martensite in low-alloy steels with different carbon content. Scr. Mater..

[17] Zhang H., Bai B., Raabe D. (2011). Superplastic martensitic Mn–Si–Cr–C steel with 900% elongation. Acta Mater..

[18] Zhang H., Zhang L., Cheng X., Bai B. (2010). Superplastic characteristic of Mn–Si–Cr alloyed ultrahigh carbon steel realized through a novel process. Acta Mater..

[19] Zhang H., Zhang L., Cheng X., Xu L., Bai B. (2010). Superplastic behavior during warm deformation of martensite in medium carbon steel. Scr. Mater..

[20] Cao Z., Wu G., Sun X., Wang C., Ponge D., Cao W. (2018). Revealing the superplastic deformation behaviors of hot rolled 0.10C5Mn2Al steel with an initial martensitic microstructure. Scr. Mater..

[21] Yang W., Jiang H., Zhou P., Shao B., Zong Y. (2025). Continuous and discontinuous dynamic recrystallization in the superplastic deformation of moderately cold-deformed Cr4Mo4Ni4V martensitic steel. J. Mater. Process. Technol..

[22] Guo Q., Liu H., Sun C., Liu H., Cao Y., Wang L., Cai X., Fu P., Wang P., Li D. (2023). Effectively improving the hardness-strength-toughness of carburized bearing steel via nanoprecipitates and fine grain structure. Mater. Sci. Eng. A.

[23] Bhattacharyya A., Subhash G., Arakere N. (2014). Evolution of subsurface plastic zone due to rolling contact fatigue of M-50 NiL case hardened bearing steel. Int. J. Fatigue.

[24] Ding Z., Guo J., Niu J., Zhou L., Zhang X., Ma X. (2024). Fabrication and mechanical properties of micro/nano-crystalline layers in M50NiL carburized steel. Mater. Des..

[25] Ji G., Li L., Qin F., Zhu L., Li Q. (2017). Comparative study of phenomenological constitutive equations for an as-rolled M50NiL steel during hot deformation. J. Alloy. Compd..

[26] Zhang Y., Yang M., Long S., Li B., Liang Y., Ma S. (2020). Effect of Initial State and Deformation Conditions on the Hot Deformation Behavior of M50NiL Steel. Materials.

[27] Yang W., Shao B., Jiang H., Zou H., Yan S., Ma Y., Tang W., Zhou P., Zong Y. (2025). Abnormal reduction in work-hardening rate of Cr4Mo4Ni4V martensitic steel induced by interface carbides suppressing boundary sliding. Mater. Sci. Eng. A.

[28] Luo B., Mao W., Zhao Y., Gao H., Ma W., Sheng S., Park N., Bhattacharjee P., Wang Q. (2025). Revisiting rapid tempering of martensitic steel: The key role of tempering time over heating rate in carbide refinement and precipitation strengthening. J. Mater. Res. Technol..

[29] Teng H., Deng Y., Dang R., Zhao H., Wei X., Dong H. (2025). Effect of carbide characteristics on abrasive wear behavior during knife honing: A comparative study of martensitic stainless steel and spring steel. J. Mater. Res. Technol..

